# Supplementary material for: Phenylpyrazolone-1,2,3-triazole Hybrids as Potent Antiviral Agents with Promising SARS-CoV-2 Main Protease Inhibition Potential
Source: Pharmaceuticals (Basel). 2023 Mar 20;16(3):463. doi: 10.3390/ph16030463 (PMC10051656; doi:10.3390/ph16030463)

Phenylpyrazolone-1,2,3-triazole hybrids: A drug design approach towards the discovery of potent antiviral agents with promising anti-SARS-CoV-2 main protease inhibition potential

Supplementary Materials

1 Chemistry 3

1.1 General click procedure for the synthesis of 1,2,3-triazole-pyrazolehybrids 6a-q 3

1.1.1 (Z)-4-(4-((4-((1-(4-Chlorophenyl)-3-methyl-5-oxo-1H-pyrazol-4(5H)-ylidene)methyl)phenoxy)methyl)-1H-1,2,3-triazol-1-yl)benzoic acid (6a) 3

1.1.2 (Z)-4-(4-((4-((3-Methyl-5-oxo-1-phenyl-1H-pyrazol-4(5H) ylidene)methyl)phenoxy)methyl)-1H-1,2,3-triazol-1-yl)benzoic acid (6b) 4

1.1.3 (Z)-4-(4-((1-(4-Acetylphenyl)-1H-1,2,3-triazol-4-yl)methoxy)benzylidene)-1-(4-chlorophenyl)-3-methyl-1H-pyrazol-5(4H)-one (6c) 4

1.1.4 (Z)-1-(4-Chlorophenyl)-4-(4-((1-(4-fluorophenyl)-1H-1,2,3-triazol-4-yl)methoxy)benzylidene)-3-methyl-1H-pyrazol-5(4H)-one (6d) 5

1.1.5 (Z)-1-(4-Chlorophenyl)-3-methyl-4-(4-((1-(4-nitrophenyl)-1H-1,2,3-triazol-4-yl)methoxy)benzylidene)-1H-pyrazol-5(4H)-one (6e) 5

1.1.6 (Z)-1-(4-Chlorophenyl)-4-(4-((1-(4-chlorophenyl)-1H-1,2,3-triazol-4-yl)methoxy)benzylidene)-3-methyl-1H-pyrazol-5(4H)-one (6f) 6

1.1.7 (Z)-1-(4-Chlorophenyl)-4-(4-((1-(3,4-dichlorophenyl)-1H-1,2,3-triazol-4-yl)methoxy)benzylidene)-3-methyl-1H-pyrazol-5(4H)-one (6g) 6

1.1.8 (Z)-1-(4-Chlorophenyl)-4-(4-((1-(3-fluoro-4-methylphenyl)-1H-1,2,3-triazol-4-yl)methoxy)benzylidene)-3-methyl-1H-pyrazol-5(4H)-one (6h) 6

1.1.9 (Z)-4-(2-(4-((4-((1-(4-Chlorophenyl)-3-methyl-5-oxo-1H-pyrazol-4(5H)-ylidene)methyl)phenoxy)methyl)-1H-1,2,3-triazol-1-yl)acetamido)benzoic acid (6i) 7

1.1.10 (Z)-2-(4-((4-((1-(4-Chlorophenyl)-3-methyl-5-oxo-1H-pyrazol-4(5H)-ylidene)methyl)phenoxy)methyl)-1H-1,2,3-triazol-1-yl)-N-(4-fluorophenyl)acetamide (6j) 7

1.1.11 (Z)-N-(4-Fluorophenyl)-2-(4-((4-((3-methyl-5-oxo-1-phenyl-1H-pyrazol-4(5H)-ylidene)methyl)phenoxy)methyl)-1H-1,2,3-triazol-1-yl)acetamide (6k)......................... 8

1.1.12 (Z)-2-(4-((4-((1-(4-Chlorophenyl)-3-methyl-5-oxo-1H-pyrazol-4(5H)-ylidene)methyl)phenoxy)methyl)-1H-1,2,3-triazol-1-yl)-N-(4-nitrophenyl)acetamide (6l) 9

1.1.13 (Z)-2-(4-((4-((1-(4-Chlorophenyl)-3-methyl-5-oxo-1H-pyrazol-4(5H)-ylidene)methyl)phenoxy)methyl)-1H-1,2,3-triazol-1-yl)-N-(3-fluoro-4-methylphenyl)acetamide (6m)... 9

1.1.14 (Z)-N-(4-Chlorobenzyl)-2-(4-((4-((1-(4-chlorophenyl)-3-methyl-5-oxo-1H-pyrazol-4(5H)-ylidene)methyl)phenoxy)methyl)-1H-1,2,3-triazol-1-yl)acetamide (6n) . 10

1.1.15 (Z)-2-(4-((4-((1-(4-Chlorophenyl)-3-methyl-5-oxo-1H-pyrazol-4(5H)-ylidene)methyl)phenoxy)methyl)-1H-1,2,3-triazol-1-yl)-N-(4-methylbenzyl)acetamide (6o) 10

1.1.16 (Z)-2-(4-((4-((1-(4-Chlorophenyl)-3-methyl-5-oxo-1H-pyrazol-4(5H)-ylidene)methyl)phenoxy)methyl)-1H-1,2,3-triazol-1-yl)-N-(4-methoxybenzyl)acetamide (6p) 11

1.1.17 (Z)-2-(4-((4-((1-(4-Chlorophenyl)-3-methyl-5-oxo-1H-pyrazol-4(5H)-ylidene)methyl)phenoxy)methyl)-1H-1,2,3-triazol-1-yl)-N-(4-fluorobenzyl)acetamide (6q) 11

2 Spectral Data of New Compounds 13

3 M^{pro} protease inhibition assay 22

4 SARS-CoV-2 Antiviral assay 22

5 Molecular dynamics simulation 23

6 The data of control drug GC-376..24

1 Chemistry

1.1 General click procedure for the synthesis of 1,2,3-triazole-pyrazolehybrids 6a-q

A solution of propargylated pyrazoles 4a-b (1 mmol) in DMSO was added with stirring to a solution of copper sulphate (0.10 g) and sodium ascorbate (0.15 g) in water (10 mL) (10 mL). The suitable azide **5a-o** (1 mmol) was then added to the reaction mixture, which was agitated at room temperature for 24-40 hours. The reaction was monitored by TLC (hexane-ethyl acetate). When the reaction was finished, the liquid was put into chilled water. The resulting precipitate was collected through filtering, washed with a saturated solution of ammonium chloride, and recrystallized from ethanol/DMF to get the desired 1,2,3-triazoles **6a-q,** all molecular characterizations are described in supplementary section.

1.1.1 *(Z)-4-(4-((4-((1-(4-Chlorophenyl)-3-methyl-5-oxo-1H-pyrazol-4(5H)-ylidene)methyl)phenoxy)methyl)-1H-1,2,3-triazol-1-yl)benzoic acid (6a)*

It was obtained as orange pellets in 86 % yield, mp: 198-199 ºC. IR (υ, cm^{-1}): 1550 (C=C), 1710 (C=O), 2930 (CH-Al), 3040 (CH-Ar). ^1H NMR (400 MHz, DMSO-d_6) δ_H = 2.36 (s, 3H, C**H**$_3$), 5.33 (s, 2H, OC**H**$_2$), 7.30 (d, 2H, *J* = 8.0 Hz, Ar-**H**), 7.47 (t, 1H, *J* = 8.0 Hz, Ar-**H**), 7.73 (d, 1H, *J* = 8.0 Hz, Ar-**H**), 7.72-7.80 (m, 4H, Ar-**H**), 7.90-7.98 (m, 5H, **H**-C=C and Ar-**H**), 9.00 (s, 1H, C**H**-1,2,3-triazole), 12.76 (s, 1H, COO**H**). ^{13}C NMR (100 MHz, DMSO-d_6): δ_C = 13.60 (**C**H$_3$), 52.75 (O**C**H$_2$); 115.59, 117.89, 120.35, 124.31, 125.38,

126.48, 127.49, 128.68, 129.28, 136.67, 137.24, 137.67, 144.46, 147.76, 149.67, 152.75, 161.89, 162.89 (Ar-**C, C**=O), 168.87 (**C**OOH). Calculated for $C_{27}H_{20}ClN_5O_4$: C, 63.10; H, 3.92; N, 13.63. Found: C, 63.29; H, 3.77; N, 13.49.

1.1.2 (Z)-4-(4-((4-((3-Methyl-5-oxo-1-phenyl-1H-pyrazol-4(5H) ylidene)methyl)phenoxy)methyl)-1H-1,2,3-triazol-1-yl)benzoic acid (6b)

It was obtained as orange pellets in 88 % yield, mp: 205-206 ºC. IR (*υ*, cm^{-1}): 1560 (C=C), 1720 (C=O), 2960 (CH-Al), 3060 (CH-Ar). ^1H NMR (400 MHz, DMSO-d_6) δ_H = 2.34 (s, 3H, C**H**$_3$), 5.37 (s, 2H, OC**H**$_2$), 7.31 (d, 2H, *J* = 8.0 Hz, Ar-**H**), 7.49 (t, 1H, *J* = 8.0 Hz, Ar-**H**), 7.70 (d, 1H, *J* = 8.0 Hz, Ar-**H**), 7.76-7.83 (m, 4H, Ar-**H**), 7.91-8.00 (m, 6H, **H**-C=C and Ar-**H**), 9.02 (s, 1H, C**H**-1,2,3-triazole), 12.88 (s, 1H, COO**H**). ^{13}C NMR (100 MHz, DMSO-d_6): δ_C = 13.64 (**C**H$_3$), 52.72 (O**C**H$_2$); 115.52, 117.80, 120.24, 124.38, 125.25, 126.39, 127.42, 128.64, 129.21, 136.52, 137.02, 137.58, 144.30, 147.84, 149.56, 152.86, 161.80, 162.92 (Ar-**C**, **C**=O), 168.46 (**C**OOH). Calculated for $C_{27}H_{21}N_5O_4$: C, 67.63; H, 4.41; N, 14.61. Found: C, 67.88; H, 4.65; N, 14.89.

1.1.3 (Z)-4-(4-((1-(4-Acetylphenyl)-1H-1,2,3-triazol-4-yl)methoxy)benzylidene)-1-(4-chlorophenyl)-3-methyl-1H-pyrazol-5(4H)-one (6c)

It was obtained as orange pellets in 90 % yield, mp: 212-213 ºC. IR (*υ*, cm^{-1}): 1565 (C=C), 1690 (C=O), 2945 (CH-Al), 3020 (CH-Ar). ^1H NMR (400 MHz, DMSO-d_6) δ_H = 2.33 (s, 3H, C**H**$_3$), 2.65 (s, 3H, COC**H**$_3$), 5.37 (s, 2H, OC**H**$_2$), 7.28 (d, 2H, *J* = 8.0 Hz, Ar-**H**), 7.46 (t, 1H, *J* = 8.0 Hz, Ar-**H**), 7.67 (d, 1H, *J* = 8.0 Hz, Ar-**H**), 7.73-7.80 (m, 4H, Ar-**H**), 7.89-7.96 (m, 5H, **H**-C=C and Ar-**H**), 9.01 (s, 1H, C**H**-1,2,3-triazole). ^{13}C NMR (100 MHz, DMSO-d_6): δ_C = 13.60 (**C**H$_3$), 26.64 (**C**H$_3$); 52.72 (O**C**H$_2$); 115.50, 117.81, 120.04, 124.39, 125.32, 126.30, 127.39, 128.62, 129.18, 136.65, 137.23, 137.70, 144.06, 146.56,

149.15, 152.66, 161.77, 162.87 (Ar-**C**, **C**=O); 194.25 (CH_3**C**=O). Calculated for $C_{28}H_{22}ClN_5O_3$: C, 65.69; H, 4.33; N, 13.68. Found: C, 65.82; H, 4.56; N, 13.89.

1.1.4 *(Z)-1-(4-Chlorophenyl)-4-(4-((1-(4-fluorophenyl)-1H-1,2,3-triazol-4-yl)methoxy)benzylidene)-3-methyl-1H-pyrazol-5(4H)-one (6d)*

It was obtained as orange pellets in 92 % yield, mp: 225-226 ºC. IR (υ, cm^{-1}): 1550 (C=C), 1690 (C=O), 2910 (CH-Al), 3040 (CH-Ar). ^{1}H NMR (400 MHz, DMSO-d_6) δ_H = 2.31 (s, 3H, C**H**$_3$), 5.33 (s, 2H, OC**H**$_2$), 7.29 (d, 2H, *J* = 8.0 Hz, Ar-**H**), 7.54 (t, 1H, *J* = 8.0 Hz, Ar-**H**), 7.74 (d, 1H, *J* = 8.0 Hz, Ar-**H**), 7.79-7.85 (m, 4H, Ar-**H**), 7.90-7.97 (m, 5H, **H**-C=C and Ar-**H**), 9.04 (s, 1H, C**H**-1,2,3-triazole). ^{13}C NMR (100 MHz, DMSO-d_6): δ_C = 13.63 (**C**H$_3$), 52.68 (O**C**H$_2$); 115.51, 117.86, 120.38, 124.78, 125.48, 126.51, 127.52, 128.81, 129.25, 136.60, 137.38, 137.46, 144.27, 146.78, 149.25, 152.58, 161.87, 162.84, 164.89 (Ar-**C**, **C**=O). Calculated for $C_{20}H_{15}ClN_2O_2$: C, 64.00; H, 3.93; N, 14.35. Found: C, 64.34; H, 3.69; N, 14.56.

1.1.5 *(Z)-1-(4-Chlorophenyl)-3-methyl-4-(4-((1-(4-nitrophenyl)-1H-1,2,3-triazol-4-yl)methoxy)benzylidene)-1H-pyrazol-5(4H)-one (6e)*

It was obtained as orange pellets in 88 % yield, mp: 232-234 ºC. IR (υ, cm^{-1}): 1545 (C=C), 1695 (C=O), 2920 (CH-Al), 3040 (CH-Ar). ^{1}H NMR (400 MHz, DMSO-d_6) δ_H = 2.36 (s, 3H, C**H**$_3$), 5.34 (s, 2H, OC**H**$_2$), 7.29 (d, 2H, *J* = 8.0 Hz, Ar-**H**), 7.48 (t, 1H, *J* = 8.0 Hz, Ar-**H**), 7.72 (d, 1H, *J* = 8.0 Hz, Ar-**H**), 7.78-7.85 (m, 4H, Ar-**H**), 7.92-7.99 (m, 5H, **H**-C=C and Ar-**H**), 9.04 (s, 1H, C**H**-1,2,3-triazole). ^{13}C NMR (100 MHz, DMSO-d_6): δ_C = 13.66 (**C**H$_3$), 52.78 (O**C**H$_2$); 115.57, 117.87, 120.15, 124.44, 125.30, 126.34, 127.48, 128.60, 129.26, 136.61, 137.09, 137.73, 144.25, 146.78, 149.24, 152.78, 161.85, 162.76 (Ar-**C**, **C**=O). Calculated for $C_{26}H_{19}ClN_6O_4$: C, 60.65; H, 3.72; N, 16.32. Found: C, 60.88; H, 3.93; N, 16.55.

1.1.6 *(Z)-1-(4-Chlorophenyl)-4-(4-((1-(4-chlorophenyl)-1H-1,2,3-triazol-4-yl)methoxy)benzylidene)-3-methyl-1H-pyrazol-5(4H)-one (6f)*

It was obtained as orange pellets in 90 % yield, mp: 203-205 ºC. IR (υ, cm^{-1}): 1570 (C=C), 1700 (C=O), 2970 (CH-Al), 3020 (CH-Ar). ^1H NMR (400 MHz, DMSO-d_6) δ_H = 2.33 (s, 3H, **C**H_3), 5.39 (s, 2H, OC**H**$_2$), 7.28 (d, 2H, J = 8.0 Hz, Ar-**H**), 7.51 (t, 1H, J = 8.0 Hz, Ar-**H**), 7.75 (d, 1H, J = 8.0 Hz, Ar-**H**), 7.81-7.87 (m, 4H, Ar-**H**), 7.93-7.98 (m, 5H, **H-**C=C and Ar-**H**), 9.00 (s, 1H, C**H**-1,2,3-triazole). ^{13}C NMR (100 MHz, DMSO-d_6): δ_C = 13.61 (**C**H_3), 52.74 (O**C**H_2); 115.58, 117.79, 120.24, 124.49, 125.33, 126.39, 127.30, 128.68, 129.21, 136.68, 137.24, 137.58, 144.10, 146.90, 149.11, 152.70, 161.79, 162.90 (Ar-**C**, **C**=O). Calculated for $C_{26}H_{19}Cl_2N_5O_2$: C, 61.92; H, 3.80; N, 13.89. Found: C, 61.82; H, 3.99; N, 13.67.

1.1.7 *(Z)-1-(4-Chlorophenyl)-4-(4-((1-(3,4-dichlorophenyl)-1H-1,2,3-triazol-4-yl)methoxy)benzylidene)-3-methyl-1H-pyrazol-5(4H)-one (6g)*

It was obtained as orange pellets in 89 % yield, mp: 244-245 ºC. IR (υ, cm^{-1}): 1530 (C=C), 1680 (C=O), 2930 (CH-Al), 3070 (CH-Ar). ^1H NMR (400 MHz, DMSO-d_6) δ_H = 2.30 (s, 3H, **C**H_3), 5.35 (s, 2H, OC**H**$_2$), 7.25 (d, 2H, J = 8.0 Hz, Ar-**H**), 7.53 (t, 1H, J = 8.0 Hz, Ar-**H**), 7.72 (d, 1H, J = 8.0 Hz, Ar-**H**), 7.82 (d, 1H, J = 8.0 Hz, Ar-**H**), 7.87-7.92 (m, 6H, **H-**C=C and Ar-**H**), 9.01 (s, 1H, C**H**-1,2,3-triazole), 9.12 (s, 1H, Ar-**H**). ^{13}C NMR (100 MHz, DMSO-d_6): δ_C = 13.65 (**C**H_3), 52.73 (O**C**H_2); 115.56, 117.88, 120.14, 124.35, 126.36, 127.14, 128.60, 129.26, 136.80, 137.19, 137.68, 144.14, 146.97, 149.18, 152.75, 161.87, 162.76 (Ar-**C**, **C**=O). Calculated for $C_{26}H_{18}Cl_3N_5O_2$: C, 57.96; H, 3.37; N, 13.00. Found: C, 57.77; H, 3.54; N, 13.23.

1.1.8 *(Z)-1-(4-Chlorophenyl)-4-(4-((1-(3-fluoro-4-methylphenyl)-1H-1,2,3-triazol-4-yl)methoxy)benzylidene)-3-methyl-1H-pyrazol-5(4H)-one (6h)*

It was obtained as orange pellets in 86 % yield, mp: 222-224 ºC. IR (υ, cm^{-1}): 1540 (C=C), 1695 (C=O), 2910 (CH-Al), 3040 (CH-Ar). ^{1}H NMR (400 MHz, DMSO-d_6) δ_H = 2.32 (s, 3H, C**H**$_3$), 2.40 (s, 3H, C**H**$_3$), 5.32 (s, 2H, OC**H**$_2$), 7.27 (d, 2H, J = 8.0 Hz, Ar-**H**), 7.51 (t, 1H, J = 8.0 Hz, Ar-**H**), 7.70 (d, 1H, J = 8.0 Hz, Ar-**H**), 7.88 (d, 1H, J = 8.0 Hz, Ar-**H**), 7.91-7.98 (m, 6H, **H-**C=C and Ar-**H**), 9.05 (s, 1H, C**H**-1,2,3-triazole), 9.15 (s, 1H, Ar-**H**). ^{13}C NMR (100 MHz, DMSO-d_6): δ_C = 13.60 (**C**H$_3$), 14.89 (**C**H$_3$), 52.71 (O**C**H$_2$); 115.64, 117.80, 120.17, 124.21, 126.30, 127.19, 128.65, 129.18, 136.87, 137.23, 137.35, 144.32, 146.83, 149.13, 152.59, 161.75, 162.98 (Ar-**C**, **C**=O). Calculated for $C_{27}H_{21}ClFN_5O_2$: C, 64.61; H, 4.22; N, 13.95. Found: C, 64.87; H, 4.49; N, 13.66.

1.1.9 *(Z)-4-(2-(4-((4-((1-(4-Chlorophenyl)-3-methyl-5-oxo-1H-pyrazol-4(5H)-ylidene)methyl)phenoxy)methyl)-1H-1,2,3-triazol-1-yl)acetamido)benzoic acid (6i)*

It was obtained as orange pellets in 90 % yield, mp: 178-179 ºC. IR (υ, cm^{-1}): 1580 (C=C), 1730 (C=O), 2950 (CH-Al), 3050 (CH-Ar), 2600-3300 (N-H). ^{1}H NMR (400 MHz, DMSO-d_6) δ_H = 2.34 (s, 3H, C**H**$_3$), 5.30 (s, 2H, NC**H**$_2$), 5.46 (s, 2H, OC**H**$_2$), 7.29-7.49 (m, 4H, Ar-**H**), 7.80-7.98 (m, 5H, Ar-**H** and **H-**C=C), 8.20-8.38 (m, 3H, Ar-**H** and C**H**-1,2,3-triazole), 8.65-8.75 (m, 2H, Ar-**H**), 11.04 (s, 1H, N**H**), 12.72 (s, 1H, COO**H**). ^{13}C NMR (100 MHz, DMSO-d_6): δ_C = 13.60 (**C**H$_3$), 52.73 (N**C**H$_2$); 61.85 (O**C**H$_2$); 107.34, 115.89, 118.70, 121.45, 124.49, 126.09, 126.98, 128.56, 130.18, 135.48, 136.87, 144.29, 144.90, 146.78, 149.87, 151.97, 162.56, 163.09, 165.67 (Ar-**C**, **C**=O), 167.34 (**C**OOH). Calculated for $C_{29}H_{23}ClN_6O_5$: C, 61.00; H, 4.06; N, 14.72. Found: C, 61.23; H, 4.29; N, 14.91.

1.1.10 *(Z)-2-(4-((4-((1-(4-Chlorophenyl)-3-methyl-5-oxo-1H-pyrazol-4(5H)-ylidene)methyl)phenoxy)methyl)-1H-1,2,3-triazol-1-yl)-N-(4-fluorophenyl)acetamide (6j)*

It was obtained as orange pellets in 90 % yield, mp: 180-181 ºC. IR (υ, cm^{-1}): 1545 (C=C), 1710 (C=O), 2940 (CH-Al), 3060 (CH-Ar), 3340 (N-H). ^{1}H NMR (400 MHz, DMSO-d_6) δ_H = 2.32 (s, 3H, C**H**$_3$), 5.35 (s, 2H, NC**H**$_2$), 5.39 (s, 2H, OC**H**$_2$), 7.17-7.27 (m, 5H, Ar-**H**), 7.48 (d, 2H, *J* = 8.0 Hz, Ar-**H**), 7.63 (d, 2H, *J* = 8.0 Hz, Ar-**H**), 7.78 (s, 1H, **H**-C=C), 7.97 (d, 2H, *J* = 8.0 Hz, Ar-**H**), 8.34 (s, 1H, C**H**-1,2,3-triazole), 8.71 (d, 1H, *J* = 4.0 Hz, Ar-**H**), 10.77 (s, 1H, N**H**). ^{13}C NMR (100 MHz, DMSO-d_6): δ_C = 13.60 (**C**H$_3$), 52.68 (N**C**H$_2$); 61.84 (O**C**H$_2$); 106.55, 115.44, 120.02, 124.01, 127.23, 129.19, 132.12, 137.61, 138.16, 142.35, 148.84, 152.69, 159.48, 161.94, 162.29, 162.91, 164.78 (Ar-**C**, **C**=O). Calculated for $C_{28}H_{22}ClFN_6O_3$: C, 61.71; H, 4.07; N, 15.42. Found: C, 61.52; H, 4.29; N, 15.62.

1.1.11 (Z)-N-(4-Fluorophenyl)-2-(4-((4-((3-methyl-5-oxo-1-phenyl-1H-pyrazol-4(5H)-ylidene)methyl)phenoxy)methyl)-1H-1,2,3-triazol-1-yl)acetamide (6k)

It was obtained as orange pellets in 92 % yield, mp: 201-203 ºC. IR (υ, cm^{-1}): 1555 (C=C), 1720 (C=O), 2960 (CH-Al), 3040 (CH-Ar), 3360 (N-H). ^{1}H NMR (400 MHz, DMSO-d_6) δ_H = 2.34 (s, 3H, C**H**$_3$), 5.33 (s, 2H, NC**H**$_2$), 5.37 (s, 2H, OC**H**$_2$), 7.17-7.29 (m, 6H, Ar-**H**), 7.47 (d, 2H, *J* = 8.0 Hz, Ar-**H**), 7.66 (d, 2H, *J* = 8.0 Hz, Ar-**H**), 7.76 (s, 1H, **H**-C=C), 7.96 (d, 2H, *J* = 8.0 Hz, Ar-**H**), 8.37 (s, 1H, C**H**-1,2,3-triazole), 8.74 (d, 1H, *J* = 4.0 Hz, Ar-**H**), 10.81 (s, 1H, N**H**). ^{13}C NMR (100 MHz, DMSO-d_6): δ_C = 13.63 (**C**H$_3$), 52.64 (N**C**H$_2$); 61.86 (O**C**H$_2$); 106.64, 115.51, 120.15, 122.22, 127.29, 129.34, 132.20, 137.69, 138.11, 142.34, 148.73, 152.46, 159.37, 161.87, 162.18, 162.85, 164.88 (Ar-**C**, **C**=O). Calculated for $C_{28}H_{23}FN_6O_3$: C, 65.87; H, 4.54; N, 16.46. Found: C, 65.63; H, 4.29; N, 16.23.

1.1.12 *(Z)-2-(4-((4-((1-(4-Chlorophenyl)-3-methyl-5-oxo-1H-pyrazol-4(5H)-ylidene)methyl)phenoxy)methyl)-1H-1,2,3-triazol-1-yl)-N-(4-nitrophenyl)acetamide (6l)*

It was obtained as orange pellets in 92 % yield, mp: 199-200 ºC. IR (υ, cm^{-1}): 1570 (C=C), 1710 (C=O), 2930 (CH-Al), 3080 (CH-Ar), 3320 (N-H). ^{1}H NMR (400 MHz, DMSO-d_6) δ_H = 2.31 (s, 3H, C**H**$_3$), 5.34 (s, 2H, NC**H**$_2$), 5.44 (s, 2H, OC**H**$_2$), 7.26-7.47 (m, 4H, Ar-**H**), 7.81-7.95 (m, 5H, Ar-**H** and **H**-C=C), 8.25-8.40 (m, 3H, Ar-**H** and C**H**-1,2,3-triazole), 8.71-8.79 (m, 2H, Ar-**H**), 11.11 (s, 1H, N**H**). ^{13}C NMR (100 MHz, DMSO-d_6): δ_C = 13.63 (**C**H$_3$), 52.79 (N**C**H$_2$); 61.82 (O**C**H$_2$); 109.54, 115.70, 119.50, 120.10, 124.05, 125.64, 126.83, 128.62, 129.25, 135.79, 137.37, 143.07, 144.94, 146.34, 149.04, 152.80, 162.34, 162.96, 165.80 (Ar-**C**, **C**=O). Calculated for $C_{28}H_{22}ClN_7O_5$: C, 58.80; H, 3.88; N, 17.14. Found: C, 58.99; H, 4.09; N, 17.33.

1.1.13 *(Z)-2-(4-((4-((1-(4-Chlorophenyl)-3-methyl-5-oxo-1H-pyrazol-4(5H)-ylidene)methyl)phenoxy)methyl)-1H-1,2,3-triazol-1-yl)-N-(3-fluoro-4-methylphenyl)acetamide (6m)*

It was obtained as orange pellets in 90 % yield, mp: 218-220 ºC. IR (υ, cm^{-1}): 1580 (C=C), 1700 (C=O), 2950 (CH-Al), 3060 (CH-Ar), 3300 (N-H). ^{1}H NMR (400 MHz, DMSO-d_6) δ_H = 2.14 (s, 3H, C**H**$_3$), 2.28 (s, 3H, C**H**$_3$), 5.35 (bs, 2H, NC**H**$_2$ and OC**H**$_2$), 7.23-7.26 (m, 4H, Ar-**H**), 7.45-7.52 (m, 3H, Ar-**H**), 7.73-7.99 (m, 3H, Ar-**H** and **H**-C=C), 8.32 (s, 1H, C**H**-1,2,3-triazole), 8.68-8.75 (m, 2H, Ar-**H**), 10.62 (s, 1H, N**H**). ^{13}C NMR (100 MHz, DMSO-d_6): δ_C = 13.61 (**C**H$_3$), 15.15 (**C**H$_3$), 52.66 (N**C**H$_2$); 61.89 (O**C**H$_2$); 110.32, 115.68, 119.52, 120.34, 121.78, 124.45, 125.60, 126.67, 128.90, 135.55, 139.50, 143.90, 145.18, 149.24, 152.45, 162.56, 163.28, 165.49 (Ar-**C**, **C**=O). Calculated for $C_{29}H_{24}ClFN_6O_3$: C, 62.31; H, 4.33; N, 15.03. Found: C, 62.52; H, 4.62; N, 15.34.

1.1.14 *(Z)-N-(4-Chlorobenzyl)-2-(4-((4-((1-(4-chlorophenyl)-3-methyl-5-oxo-1H-pyrazol-4(5H)-ylidene)methyl)phenoxy)methyl)-1H-1,2,3-triazol-1-yl)acetamide (6n)*

It was obtained as orange pellets in 89 % yield, mp: 224-225 ºC. IR (*υ*, cm^{-1}): 1560 (C=C), 1700 (C=O), 2930 (CH-Al), 3080 (CH-Ar), 3320 (N-H). ^{1}H NMR (400 MHz, DMSO-d_6) δ_H = 2.32 (s, 3H, C**H**$_3$), 4.32 (s, 2H, NHC**H**$_2$), 5.32 (s, 2H, NC**H**$_2$), 5.33 (s, 2H, OC**H**$_2$), 7.25-7.31 (m, 4H, Ar-**H**), 7.39-7.47 (m, 4H, Ar-**H**), 7.76 (s, 1H, **H**-C=C), 7.96 (s, 2H, Ar-**H**), 8.27 (s, 1H, C**H**-1,2,3-triazole), 8.69 (s, 2H, Ar-**H**), 8.88 (s, 1H, N**H**). ^{13}C NMR (100 MHz, DMSO-d_6): δ_C = 13.63 (**C**H$_3$), 42.34 (NH**C**H$_2$); 52.54 (N**C**H$_2$); 61.81 (O**C**H$_2$); 106.87, 115.41, 120.24, 121.69, 127.35, 128.67, 132.03, 137.42, 137.89, 142.30, 148.25, 152.43, 156.87, 161.93, 162.43, 164.75 (Ar-**C**, **C**=O). Calculated for $C_{29}H_{24}Cl_2N_6O_3$: C, 60.53; H, 4.20; N, 14.60. Found: C, 60.38; H, 4.44; N, 14.87.

1.1.15 *(Z)-2-(4-((4-((1-(4-Chlorophenyl)-3-methyl-5-oxo-1H-pyrazol-4(5H)-ylidene)methyl)phenoxy)methyl)-1H-1,2,3-triazol-1-yl)-N-(4-methylbenzyl)acetamide (6o)*

It was obtained as orange pellets in 89 % yield, mp: 229-230 ºC. IR (*υ*, cm^{-1}): 1550 (C=C), 1710 (C=O), 2900 (CH-Al), 3090 (CH-Ar), 3310 (N-H). ^{1}H NMR (400 MHz, DMSO-d_6) δ_H = 2.17 (s, 3H, C**H**$_3$), 2.30 (s, 3H, C**H**$_3$), 4.29 (s, 2H, NHC**H**$_2$), 5.35 (s, 2H, NC**H**$_2$), 5.38 (s, 2H, OC**H**$_2$), 7.23-7.28 (m, 3H, Ar-**H**), 7.33-7.44 (m, 5H, Ar-**H**), 7.79 (s, 1H, **H**-C=C), 7.92 (bs, 2H, Ar-**H**), 8.34 (s, 1H, C**H**-1,2,3-triazole), 8.73 (s, 2H, Ar-**H**), 8.93 (s, 1H, N**H**). ^{13}C NMR (100 MHz, DMSO-d_6): δ_C = 13.60 (**C**H$_3$), 15.29 (**C**H$_3$), 42.41 (NH**C**H$_2$); 52.50 (N**C**H$_2$); 62.13 (O**C**H$_2$); 106.67, 115.62, 120.34, 121.74, 127.42, 128.54, 132.32, 137.23, 137.65, 142.12, 148.34, 152.13, 156.56, 162.08, 162.87, 164.90 (Ar-**C**,

C=O). Calculated for $C_{30}H_{27}ClN_6O_3$: C, 64.92; H, 4.90; N, 15.14. Found: C, 65.23; H, 4.63; N, 15.63.

1.1.16 (Z)-2-(4-((4-((1-(4-Chlorophenyl)-3-methyl-5-oxo-1H-pyrazol-4(5H)-ylidene)methyl)phenoxy)methyl)-1H-1,2,3-triazol-1-yl)-N-(4-methoxybenzyl)acetamide (6p)

It was obtained as orange pellets in 92 % yield, mp: 208-209 ºC. IR (υ, cm^{-1}): 1570 (C=C), 1690 (C=O), 2950 (CH-Al), 3060 (CH-Ar), 3290 (N-H). ^{1}H NMR (400 MHz, DMSO-d_6) δ_H = 2.34 (s, 3H, C**H**$_3$), 3.68 (s, 3H, C**H**$_3$), 4.35 (s, 2H, NHC**H**$_2$), 5.30 (s, 2H, NC**H**$_2$), 5.33 (s, 2H, OC**H**$_2$), 7.27-7.38 (m, 4H, Ar-**H**), 7.42-7.49 (m, 4H, Ar-**H**), 7.76 (s, 1H, **H-**C=C), 7.88 (bs, 2H, Ar-**H**), 8.31 (s, 1H, C**H**-1,2,3-triazole), 8.78 (s, 2H, Ar-**H**), 8.79 (s, 1H, N**H**). ^{13}C NMR (100 MHz, DMSO-d_6): δ_C = 13.66 (**C**H$_3$), 42.53 (NH**C**H$_2$); 52.41 (N**C**H$_2$); 55.46 (O**C**H$_3$), 62.28 (O**C**H$_2$); 106.87, 115.89, 120.59, 121.79, 128.40, 129.82, 133.56, 138.76, 143.34, 147.78, 152.54, 156.78, 162.23, 162.57, 164.76 (Ar-**C**, **C**=O). Calculated for $C_{30}H_{27}ClN_6O_4$: C, 63.10; H, 4.77; N, 14.72. Found: C, 63.36; H, 4.59; N, 14.95.

1.1.17 (Z)-2-(4-((4-((1-(4-Chlorophenyl)-3-methyl-5-oxo-1H-pyrazol-4(5H)-ylidene)methyl)phenoxy)methyl)-1H-1,2,3-triazol-1-yl)-N-(4-fluorobenzyl)acetamide (6q)

It was obtained as orange pellets in 89 % yield, mp: 245-255 ºC. IR (υ, cm^{-1}): 1570 (C=C), 1690 (C=O), 2910 (CH-Al), 3080 (CH-Ar), 3320 (N-H). ^{1}H NMR (400 MHz, DMSO-d_6) δ_H = 2.51 (bs, 3H, C**H**$_3$ overlap with DMSO-d_6), 4.31 (s, 2H, NHC**H**$_2$), 5.19 (s, 2H, NC**H**$_2$), 5.30 (s, 2H, OC**H**$_2$), 7.16-7.32 (m, 9H, Ar-**H**), 7.88 (bs, 3H, Ar-**H** and **H-**C=C), 8.28 (s, 1H, C**H**-1,2,3-triazole), 8.85 (s, 1H, Ar-**H**), 9.88 (s, 1H, N**H**). ^{13}C NMR (100 MHz, DMSO-d_6): δ_C = 13.69 (**C**H$_3$), 42.26 (NH**C**H$_2$); 52.69 (N**C**H$_2$); 61.77 (O**C**H$_2$);

106.34, 115.72, 120.23, 122.45, 126.78, 129.54, 132.42, 137.55, 138.03, 142.19, 148.67, 152.87, 158.37, 162.06, 162.90, 165.31 (Ar-**C**, **C**=O). Calculated for $C_{29}H_{24}ClFN_6O_3$: C, 62.31; H, 4.33; N, 15.03. Found: C, 62.52; H, 4.56; N, 15.28.

2 Spectral Data of New Compounds

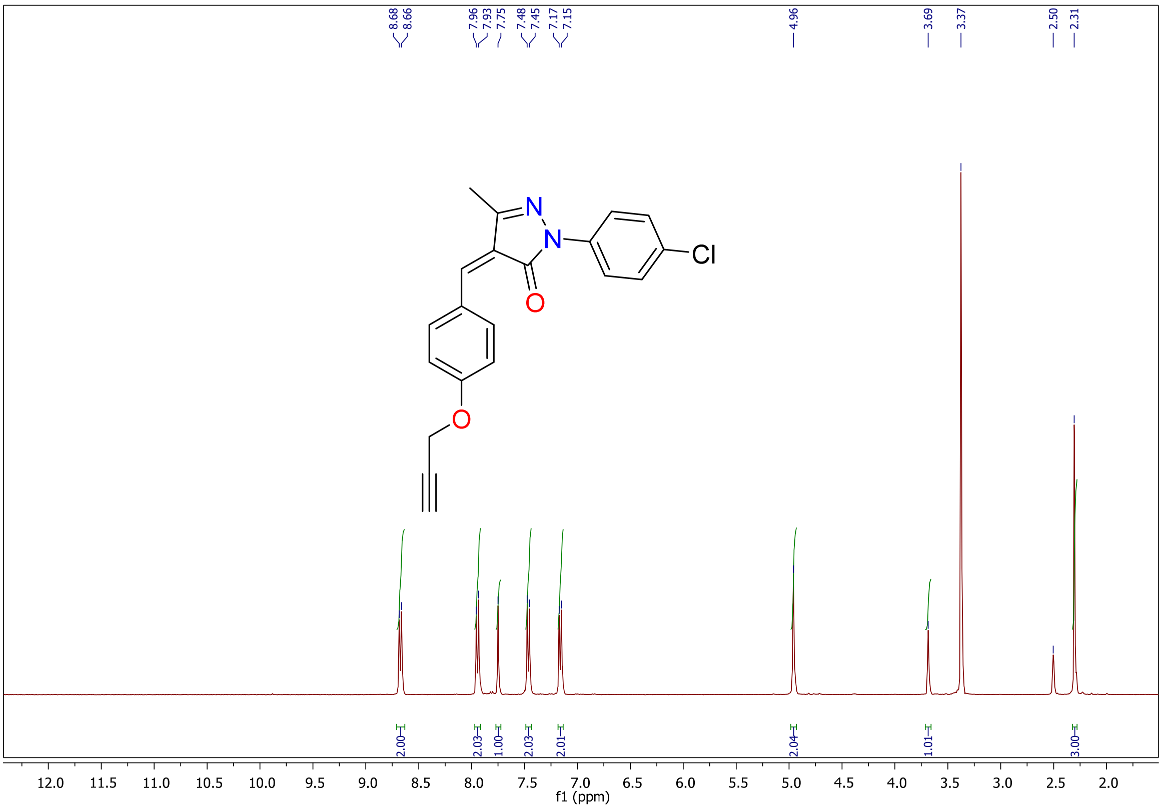

Figure S1: ^{1}H NMR spectrum of compound **4b**.

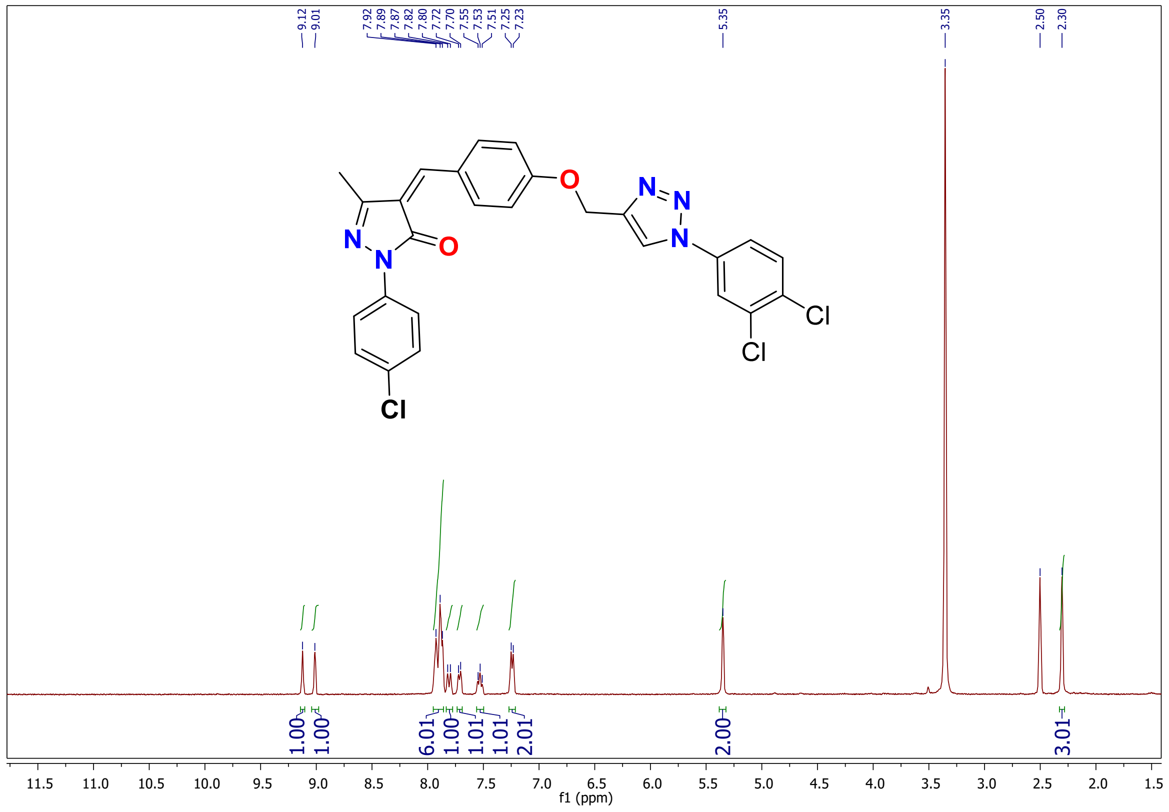

Figure S2: ^{1}H NMR spectrum of compound **6g**.

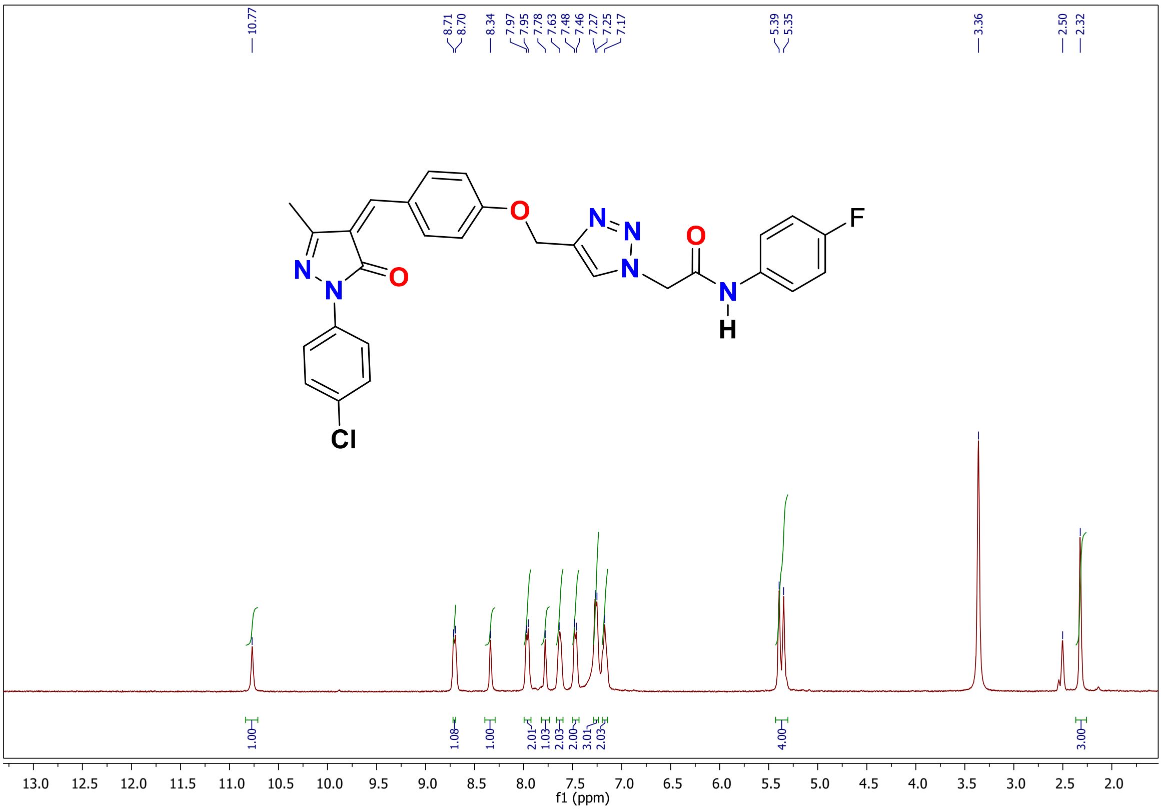

Figure S3: ^{1}H NMR spectrum of compound **6j**.

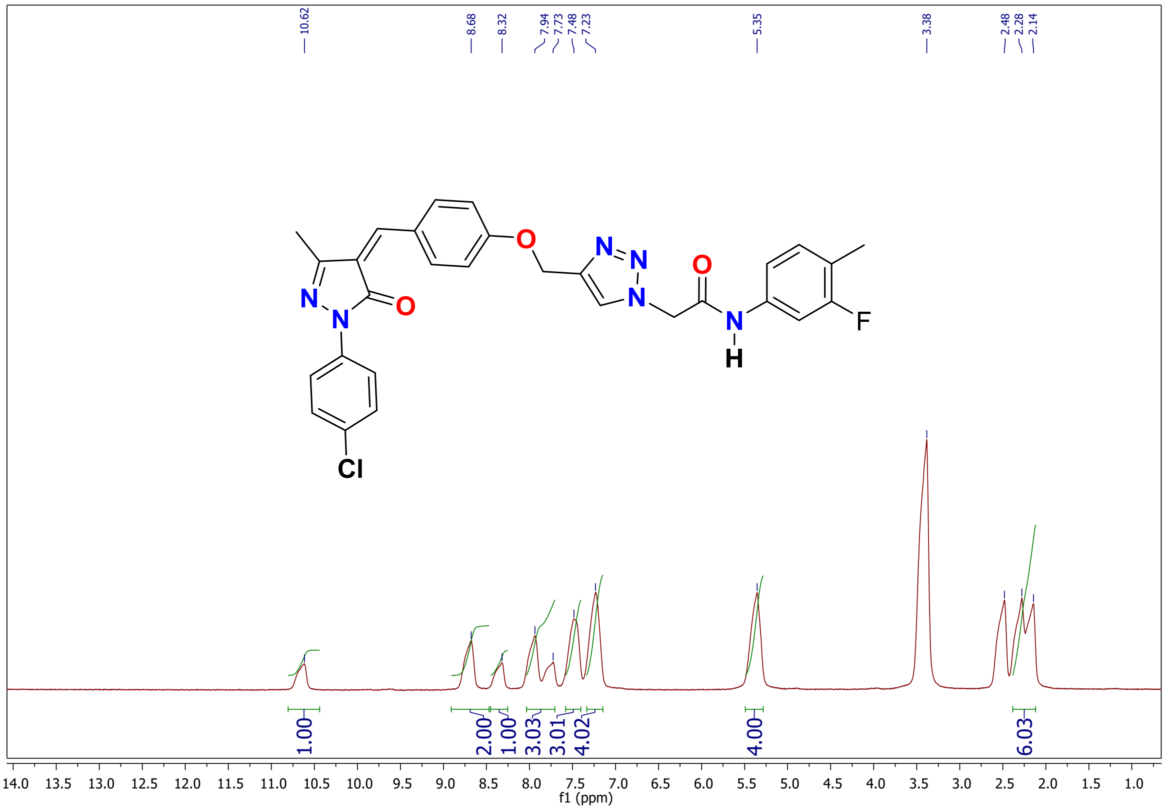

Figure S4: ^{1}H NMR spectrum of compound **6m**.

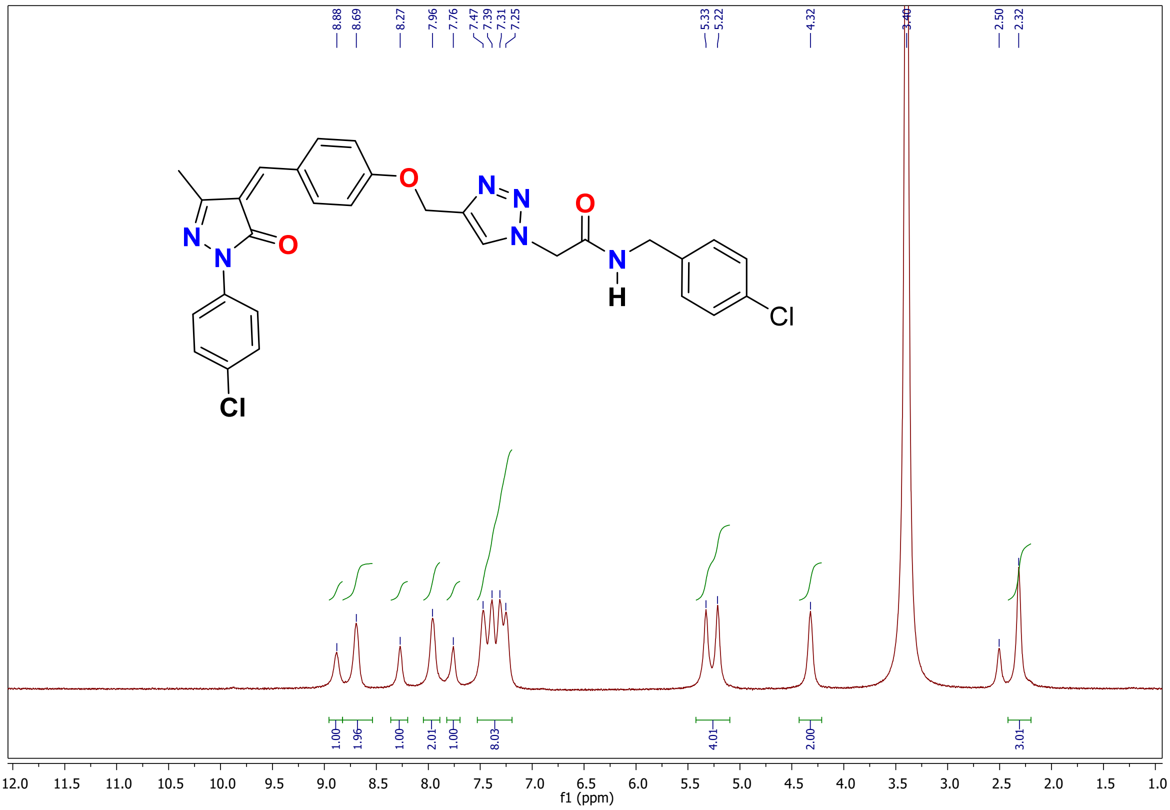

Figure S5: ^{1}H NMR spectrum of compound **6n**.

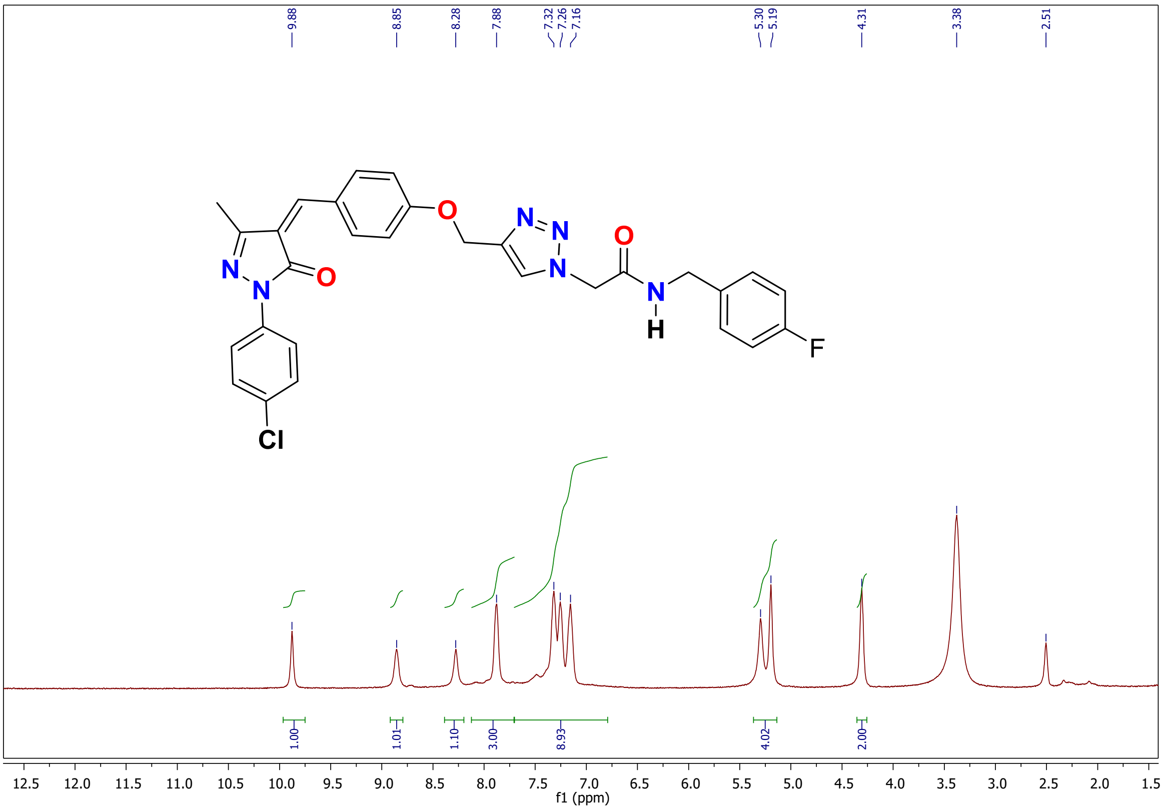

Figure S6: ^{1}H NMR spectrum of compound **6q**.

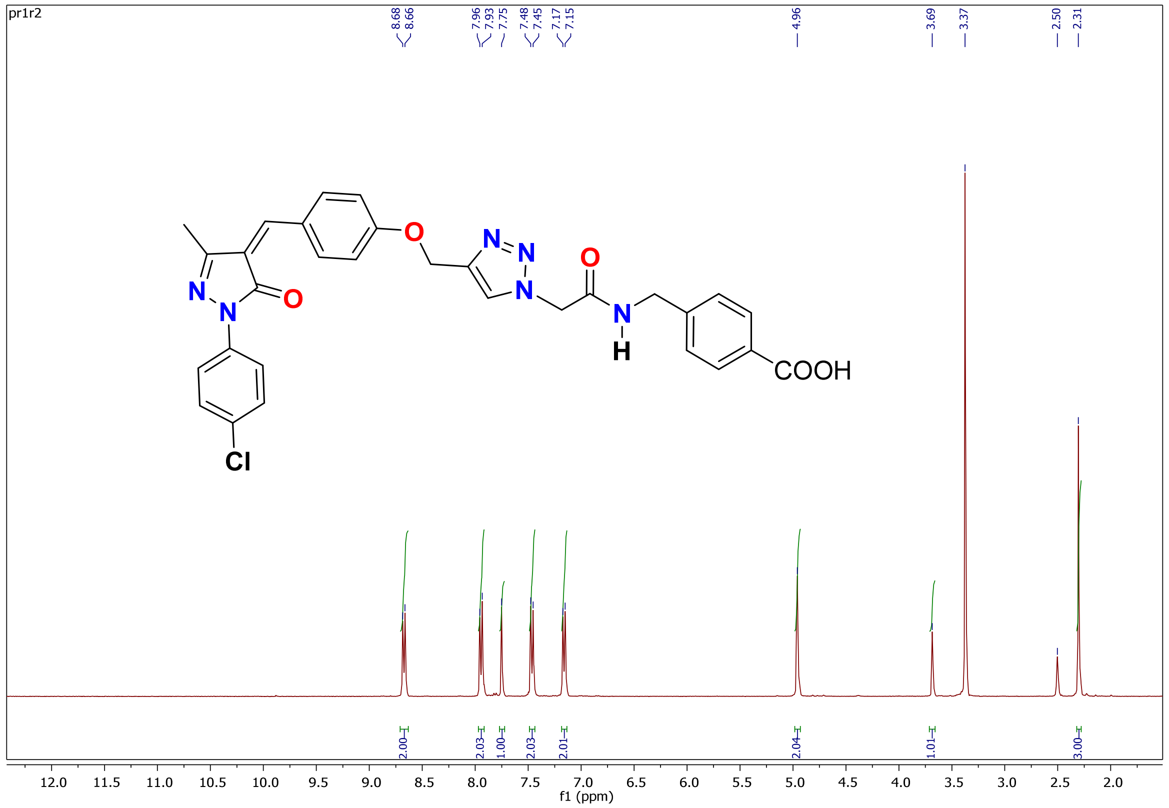

Figure S7: ^{1}H NMR spectrum of compound **6i**.

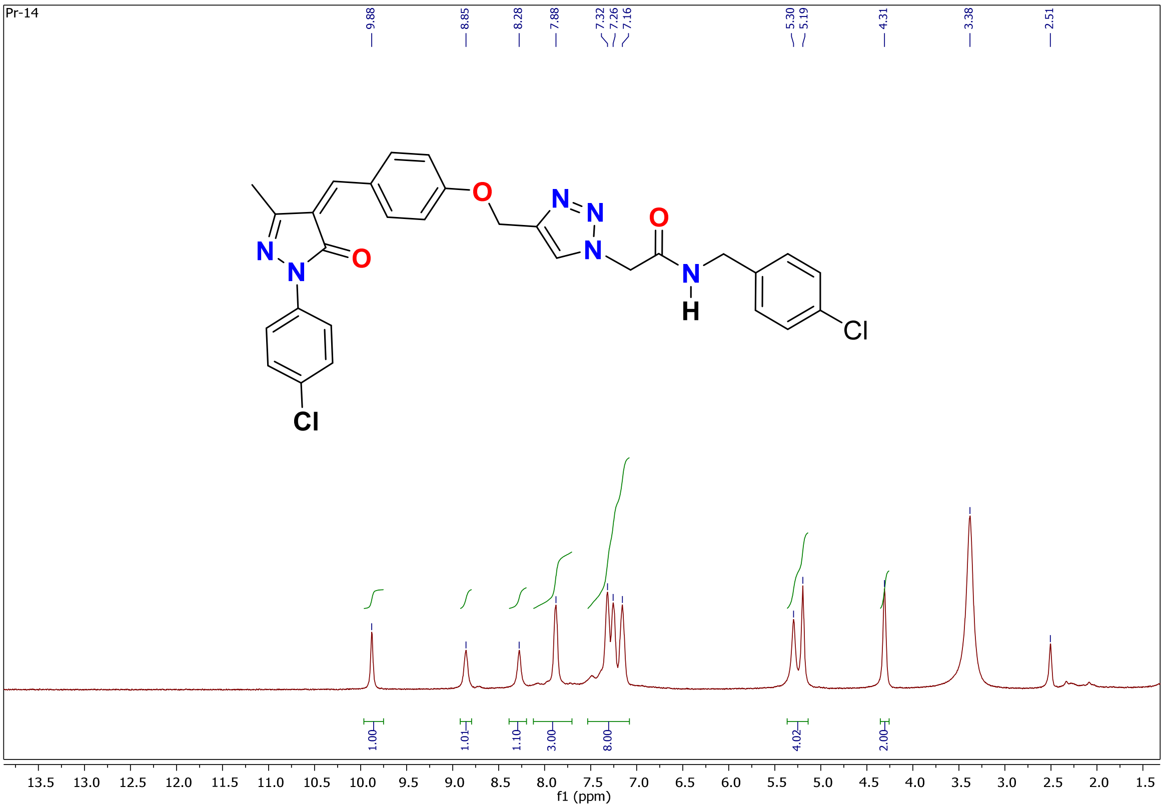

Figure S8: ^{1}H NMR spectrum of compound **6n**.

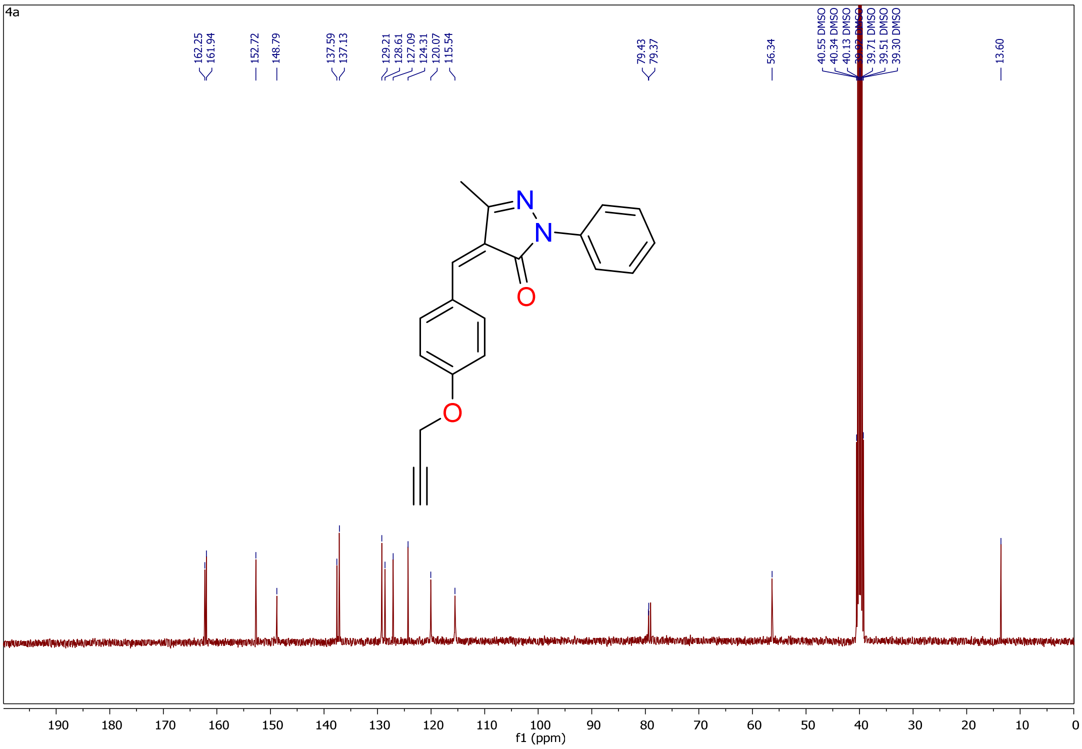

Figure S9: ^{13}C NMR spectrum of compound **4a**.

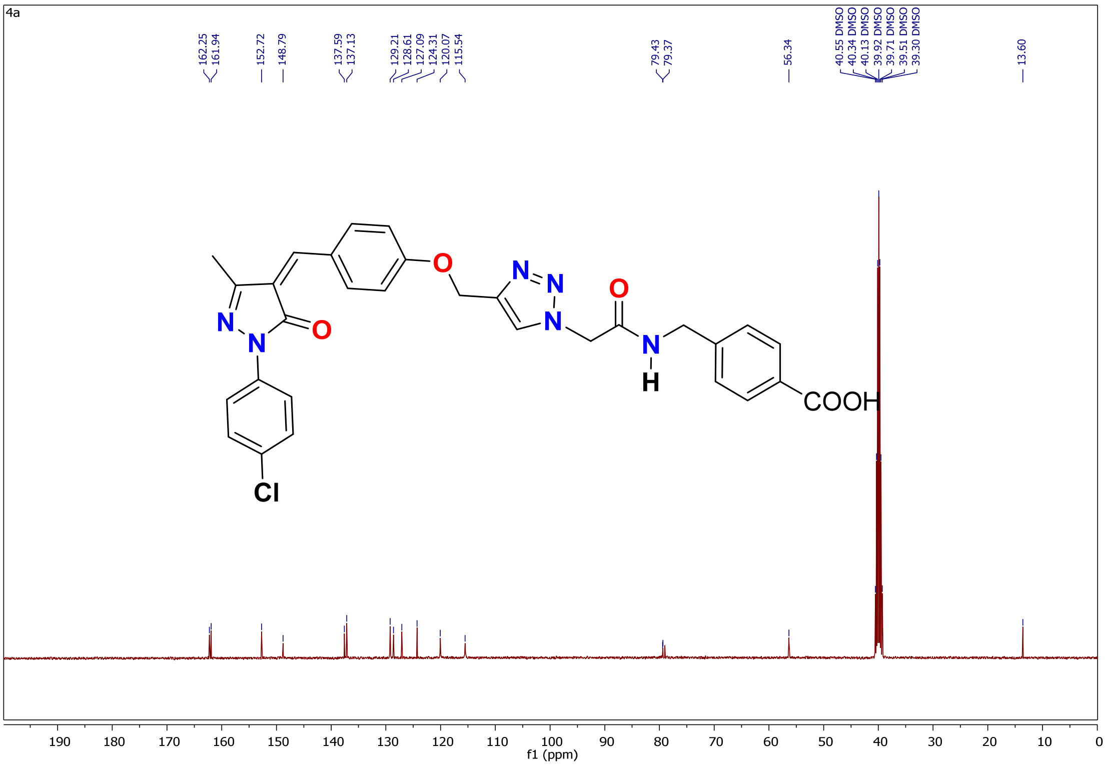

Figure S10: ^{13}C NMR spectrum of compound **6i**.

3 M^{pro} protease inhibition assay

The COV2-SARS-CoV-2 protease enzyme assay [52,53] was described in the manufacturing protocol (BPS Bioscience). In summary, it was performed by adding 30 µl of SARS-CoV-2 protease and 10 µl of compound diluted in 1× assay buffer. Then Incubated for 30 min at room temperature. The reaction began with the addition of 10 µl of the diluted substrate, which is a M^{pro} self-quenching 14-mer fluorogenic (FRET) peptide with a cleavage site indicated by an arrow ((Dabcyl-KTSAVLQ↓SGFRKME-Edans) (BPS Bioscience) using a counter-screen assay for the quenching of inhibitors' fluorescence effect. Then Incubated at room temperature overnight and the plate was sealed with the plate sealer. The fluorescence intensity was measured in a microtiter plate-reading fluorimeter capable of excitation at a wavelength of 360 nm and detection of emission at a wavelength of 460 nm. The fluorescence intensity can also be measured kinetically. The "Blank" value is subtracted from all other values. The experiment was carried out three times to acquire the IC_{50} value, and the error from the global fit with varied hill slope.

4 SARS-CoV-2 Antiviral assay

To assess the effect of different target chemical treatments on SARS-CoV-2 viral load (SARS-CoV-2 isolate EGY/WAT-2 VACCERA), a Real-Time PCR test was used to detect SARS-CoV-2 viral RNA [54,55]. Total RNA was extracted according to the manufacturer's instructions using the genesig® Coronavirus SARS-CoV-2 Real-Time PCR Assay kit (Primer design TM Ltd, Southampton, United Kingdom). This kit includes a master mix, primers, and probe for reverse transcription of extracted RNA

as well as Real-Time PCR for SARS-CoV-2 detection. The assay was carried out using the Rotor-Gene Q instrument (Qiagen, Germany) under the following amplification conditions: 10 minutes of reverse transcription at 55 °C, 2 minutes of initial activation at 95 °C, 45 cycles of 10 seconds denaturation at 95 °C and 60 seconds annealing and extension at 60 °C, and 45 cycles of annealing and extension at 60 °C.

5 Molecular dynamics simulation

MD simulations were performed utilising templates from the 3D structure crystal of SARS-COV 19 Mpro protease (PDB ID: 5R80) complexed with the target active compound **6i** vs the reference bound drug. The simulation was run with the Desmond version 3.8 software and the OPLS2005 forcefield [61]. The initial docked model structure was refined using the protein preparation wizard in Maestro (Schrödinger, LLC) and placed into TIP3P water molecules solvated with 0.15 M NaCl. After minimization and relaxation of the model, the production molecular dynamics phase was performed for three independent 100 ns simulations in an isothermal-isobaric (NpT) ensemble at 300 K and 1 bar using Langevin dynamics. Long-range electrostatic interactions were computed using the Smooth Particle Mesh Ewald method. All system setups were performed using Maestro. Trajectory coordinates were recorded every 100 ps. The simulation trajectories were then analyzed for the sampled conformations in all structural models. The obtained trajectory was processed utilizing the AMBER11 tool ptraj for the calculations of RMSD and PMSF for protein RMSD. All molecular figures were generated using PyMOL (Schrödinger, LLC), see supplementry data.

6 The data of control drug GC-376

Researcher : Dr.Hany Emary email: jan_25_misr@yahoo.com *mob.* +966534391522

Assay : COV-3CL protease assay

Samples : 01 compounds.

Cell line : ---

Reference : ---

Lab Report I

ser	Compound		Results	
	code	MW g/mol	COV-3CL protease IC50 uM	SD ±
***	GC-376	507.53	12.85	0.74

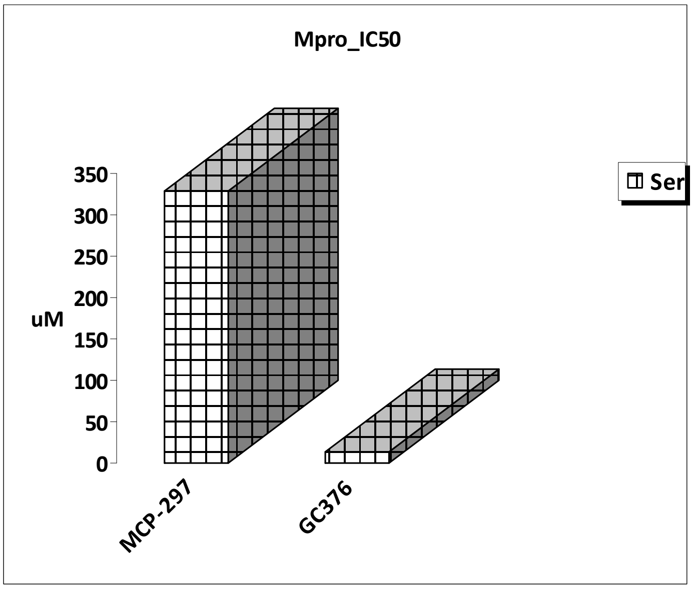

Detailed results

code	IC50	conc. uM	log conc	%inh	T2	T1	ΔT	RFU2	RFU1	ΔRFU	slope	K.Activity
GC376		1000	3	91	30	0	30	8.63	0	8.63	3.3333	10.3561
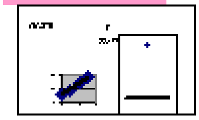		100	2	73	30	0	30	26.54	0	26.54	3.3333	31.8483
		10	1	42	30	0	30	58.11	0	58.11	3.3333	69.7327
		1	0	28	30	0	30	72.03	0	72.03	3.3333	86.4369
EC				0	30	0	30	100	0	100	3.3333	120
	[redacted]		[redacted]		[redacted]		[redacted]		[redacted]			[redacted]

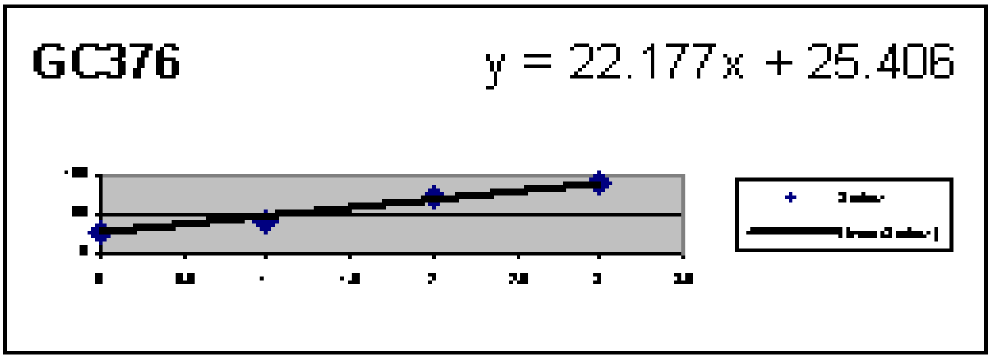

Supplement: Supplementary file 1 [file pharmaceuticals-16-00463-s001.zip › pharmaceuticals-2216659-supplementary.pdf]
